# Rare catastrophic events drive population dynamics in a bat species with negligible senescence

**DOI:** 10.1038/s41598-017-06392-9

**Published:** 2017-08-04

**Authors:** Toni Fleischer, Jutta Gampe, Alexander Scheuerlein, Gerald Kerth

**Affiliations:** 1 Applied Zoology and Conservation, Zoological Institute, University of Greifswald, Johann, Sebastian Bach-Strasse 11/12, 17487 Greifswald, Germany; 2 Statistical Demography, Max Planck Institute for Demographic Research, Konrad-Zuse-Str., 1 D-18057 Rostock, Germany; 3 Evolutionary Biodemography, Max Planck Institute for Demographic Research, Konrad-Zuse-Str., 1 D-18057 Rostock, Germany

## Abstract

Bats are remarkably long-lived with lifespans exceeding even those of same-sized birds. Despite a recent interest in the extraordinary longevity of bats very little is known about the shape of mortality over age, and how mortality rates are affected by the environment. Using a large set of individual-based data collected over 19 years in four free-ranging colonies of Bechstein’s bats (*Myotis bechsteinii*), we found no increase in the rate of mortality and no decrease in fertility demonstrating no senescence until high ages. Our finding of negligible senescence is highly unusual for long-lived mammals, grouping Bechstein’s bats with long-lived seabirds. The most important determinant of adult mortality was one particular winter season, which affected all ages and sizes equally. Apart from this winter, mortality risk did not differ between the winter and the summer season. Colony membership, a proxy for local environmental conditions, also had no effect. In addition to their implications for understanding the extra-ordinary longevity in bats, our results have strong implications for the conservation of bats, since rare catastrophic mortality events can only be detected in individual based long-term field studies. With many bat species globally threatened, such data are crucial for the successful implementation of conservation programs.

## Introduction

Bats live substantially longer than non-flying mammals of similar body size^1^ and group with birds in a regression of longevity on body mass^2^ It is generally assumed that compared to their non-flying counterparts, species capable of active flight are better able to avoid predators, and are therefore less prone to environmentally driven mortality^3^. This low rate of mortality may have ultimately led to the evolution of longer lifespans by increasing the allocation of resources into somatic maintenance^3, 4^. On the other hand, the evolution of the ability to fly might itself have required adaptations that, as a side effect, promote long lifespans^5^. For example, active flight requires physiological machinery that is able to generate high workloads, yet minimizes the molecular damage that eventually accrues^6^. But a volant lifestyle and its many implications may be only part of the story. Evidence from comparative studies of birds and mammals suggests that bats, in particular species of the temperate zone that typically hibernate for several months, surpass even birds with respect to longevity^7^. For example, members of the bat family Vespertilionidae, which typically have a body mass of between three and 40 g, have an average lifespan that is far longer than would be predicted by their body mass. Notably, a male Brandt’s bat (*Myotis brandtii*) with a body mass of less than 10 g was found to have lived 41 years in the wild^8^. In contrast, the longest-lived member of the Trochilidae, a bird family with a mass range similar to that of Vespertilionid bats, has a maximum lifespan of only 14 years^9^.

How this extraordinary longevity has evolved in some taxa of bats is so far unknown. Healy *et al*.^7^ have hypothesized that because of energetic and biomechanical requirements of flight, female bats can raise only small litters^10^. Therefore, there are few options for maximizing fitness other than an increase of lifetime opportunities for reproduction via an expanded lifespan. On the other hand, bats’ metabolic rates can vary considerably during the day and over the year. Because many species use torpor during roosting, whereby their metabolic rate can drop to one-tenth their normal rate^11^, early gerontologists attributed the long lifespan of bats exclusively to this mechanism of energy saving during unfavorable food conditions^12^. Recent studies have confirmed that hibernating bats surpass even birds with respect to longevity^7^ but also no-hibernating bats live unusually long^1^.

Despite their small body size, the low annual reproductive output of bats puts them at the ‘slow’ end of the slow-fast continuum of mammalian life histories with high adult survival and long generation times, together with large mammals such as elephants and primates^13^. We would therefore expect that senescence would scarcely be detectable, and if at all, only at very high ages, when age-specific mortality would increase while fertility would decrease^14, 15^. Since most hibernating bats have only one offspring per year, increasing annual reproductive output by an additional young would increase investment by 100 percent. On the other hand, fitness could also be maximized by an increased reproductive lifespan, which is achievable using fewer resources. Increasing reproductive lifespan would in turn lead to selection on high rates of adult survival^16^. This strategy would make bat populations extremely sensitive to fluctuations in adult survival rate^17^ and place them at high extinction risk if adult survival were compromised^18^.

Our knowledge of the factors that affect adult survival in bats is still minimal. Although a number of population studies have been conducted (see review by O’Shea *et al*.^19^), many of these studies were based on occasional recoveries of marked individuals only. To accurately estimate the effects of individual traits, age, season, or environmental fluctuations between years, studies that follow individuals over their entire lifespan are needed^20^. While a few such studies on bats exist^21–25^, including one 16-year mark-recapture study on little brown bats (*Myotis lucifugus*) by Frick *et al*.^26^, most studies did not last long enough or were not fine-scaled enough to be able to test the effects on bat mortality of age or of fluctuations in adult survival caused by rare catastrophic events.

Here, we report the results of a detailed analysis of adult mortality and fertility that draws upon a unique set of individual long-term data over 19 years on four free-ranging colonies of Bechstein’s bats^27, 28^. This dataset is comprised of the individual life histories of 248 female bats with known age. Bechstein’s bats are long-lived, and have a maximum observed lifespan of 21 years in the wild^29^. While the species is widely distributed across Europe, levels of concern about its conservation are high, and the species is listed as near threatened by the IUCN^30^. The species’ current stronghold is in central Europe, nevertheless even there, populations typically occur in low densities^30^. During spring and summer, 15–40 adult females form maternity colonies, where they give birth to up to one offspring per year^31^. For the autumn mating season, the bats move from their summer roosts to swarming sites, such as caves and mines. The bats then hibernate in these sites from November until mid-April^28^. The adult males remain solitary after their first hibernation. As dispersal events between different colonies are extremely rare, estimated to be approximately one female in five generations, each colony can be treated as an ecologically and demographically independent replicate^28, 32^.

Here, we studied adult survival, the demographic rate with the highest impact on population growth rate^17^, and how it is affected by season and age. The abovementioned study by Frick *et al*.^26^ found a high impact of summer precipitation on adult survival in one North-American species of the genus Myotis, whereas two studies on European bats belonging to other genera suggest no difference in mortality between summer and winter season^21, 33^. Therefore, we explored the influence of the season on mortality in Bechstein’s bats, with no a priori prediction regarding differences in mortality between the seasons. We predicted that for Bechstein’s bats mortality would remain at low levels throughout the longest part of adult lifespan, and that fertility would remain stable until old ages to compensate for the low annual reproduction rate of one offspring.

## Results

### Descriptive Statistics

**Figure 1:**
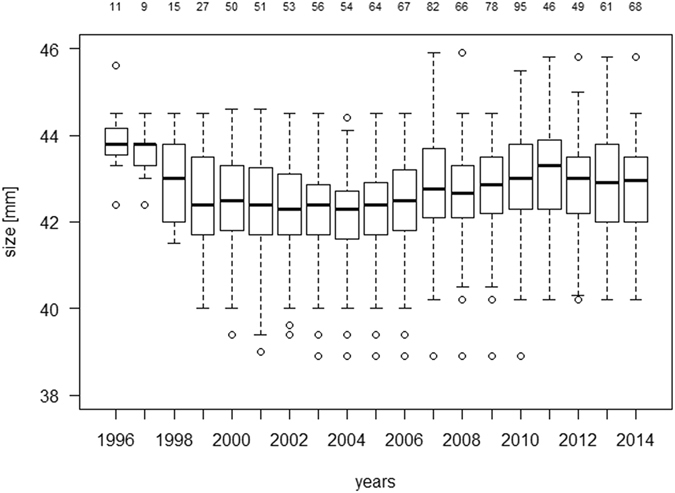
Distribution of forearm length in all four bat colonies over the study period. The numbers above the boxplots denote the number of adult animals in each year.

**Figure 2:**
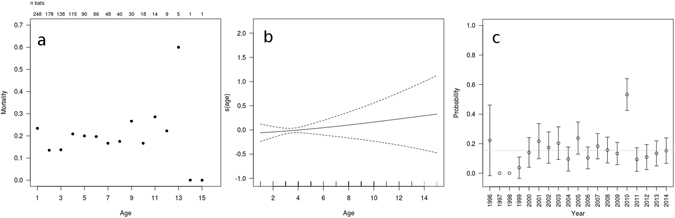
(**a**) Observed annual probability of death in each age class. (**b**) The corresponding estimation result for the hazard regression from model 7 from Table 1 with age as a smooth function on logit scale ± 2 s.e., and (**c**) environmental fluctuations via variable *year* (age and size fixed at their medians. Dashed horizontal line indicates mean; 95% confidence intervals added).

Of the 248 animals that were analysed, 180 died during the 19-year study period, and 68 were still alive at the end of the study. The forearm lengths of the bats ranged from 38.9 to 45.9 mm, and fluctuated over the study period with no definite trend discernible (Fig. 1). The minimum age at death was one year (that is, the animal did not survive another year after surviving its first hibernation). The maximum observed age was 15 years, and this animal was still alive at the end of study. The maximum observed age at death was 13 years. Figure 2a shows the mortality for each age class until age 15. These values were calculated by dividing the number of deaths in the one age class by the number of animals alive at the beginning of the age class.

### Model Building

To investigate the relative impact of age, environment, and individual body size (forearm length) on mortality, several models were run and compared. Table 1 summarizes the results for annual mortality. The variable *year* was a factor that controlled for different levels of mortality across the years (mid-April to mid-April), while *colony* was a factor that controlled for possible differences in the local habitat of the four colonies. Age and size were allowed to enter the models as smooth functions *s(age)* and *s(size)* to capture the potential nonlinear impacts of the two variables.

**Table 1:** Summary of discrete-time hazard regression models. *year* and *colony* enter as factors, while *age* and *size* are modelled as smooth functions. The last three columns give the effective degrees of freedom (edf) for the model, the deviance, and the resulting AIC, respectively.

Model	Terms	edf	Deviance	AIC
1	intercept only	1	975.76	977.76
2	*Year*	19	880.03	918.03
3	*year* + *colony*	22	878.15	922.15
4	*year* + *s*(*age*)	20.15	879.64	919.94
5	*year* + *s*(*age*) + *colony*	23.04	877.97	929.04
6	*year* + *s*(*size*)	21.18	870.33	912.68
7	*year* + *s*(*age*) + *s*(*size*)	22.36	869.23	913.95

Fluctuations in mortality due to overall environmental effects (variable *year*) led to the largest reductions in deviance, and were thus the strongest predictors of variations in mortality; while *colony*, as a proxy for local environmental effects, had no statistically significant impact. When *age* and *size* were additionally incorporated, both via smooth functions, Model 6, which included *year* and *size* only, showed the minimal value for the AIC, but Model 7, which additionally includes *age* generated almost the same results. From the perspective of achieving parsimony, Model 6, which described mortality as independent of *age*, certainly is preferred. However, we also looked at the results of Model 7, which includes *age*.

**Figure 3:**
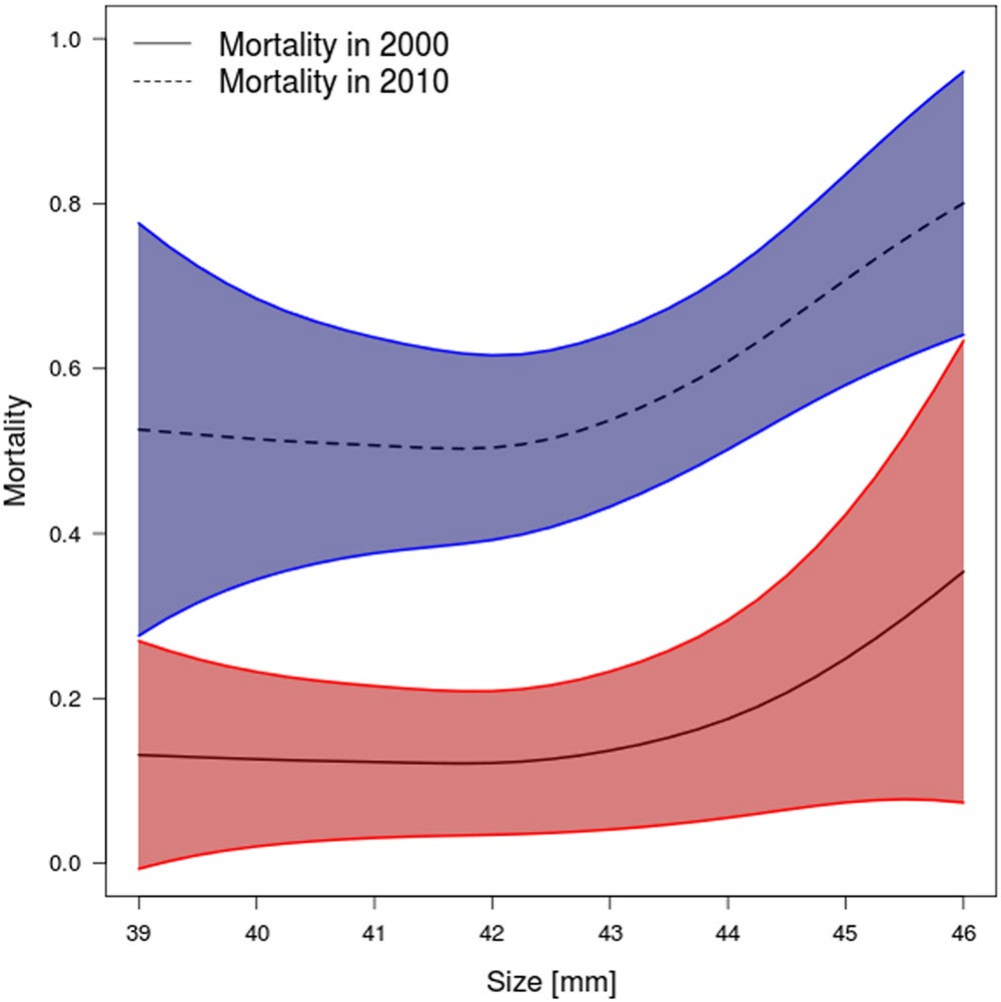
Mortality increase with forearm length (for environmental conditions fixed at year =2000 and 2010) as estimated from Model 6, 95% confidence intervals added.

Figure 2b shows the estimated function *s(age)* from the hazard regression, on a logit scale, while Fig. 2c shows the impact of different years on mortality. In 1997 and 1998 no deaths were observed; hence, the estimated probabilities were zero. Our finding that the factor *year* was important is mostly attributable to the year 2010, in which the probability of mortality was about three times higher than average; and to two additional benign years, 1999 and 2014. Because only a small number of bats survived more than 11 years, the slow increase of *s(age)* at the right end of the age distribution (Fig. 2b) was accompanied by very wide confidence intervals, and thus did not support the assumption that mortality was age dependent. By contrast, the findings for *s*(*size*) indicated that mortality was not affected by forearm lengths up to about the median size, but increased roughly linearly thereafter (Fig. 3). In summary, these results supported the initial choice of Model 6. Figure 3 shows the impact of size on mortality in this final model (*p* = 0.0352), both for the year 2000, which represented average conditions, and for the year 2010, when conditions were extreme. The effect of size was indistinguishable in normal years and in the extreme year 2010.

Since each year comprised two different ‘seasons’ of the bats’ annual life cycle, we extended the analysis and performed discrete-time hazard regression, whereby the intervals were split into ‘summer’ and ‘winter’ periods. In particular, we wanted to investigate whether there was a consistent pattern of seasonal mortality. The environmental conditions were modelled via a factor for *year* (the same for both seasons) and an additional two-level factor *season* that distinguished between the two periods of a year. The age variable in the winter season was approximated by adding 0.5.

**Figure 4:**
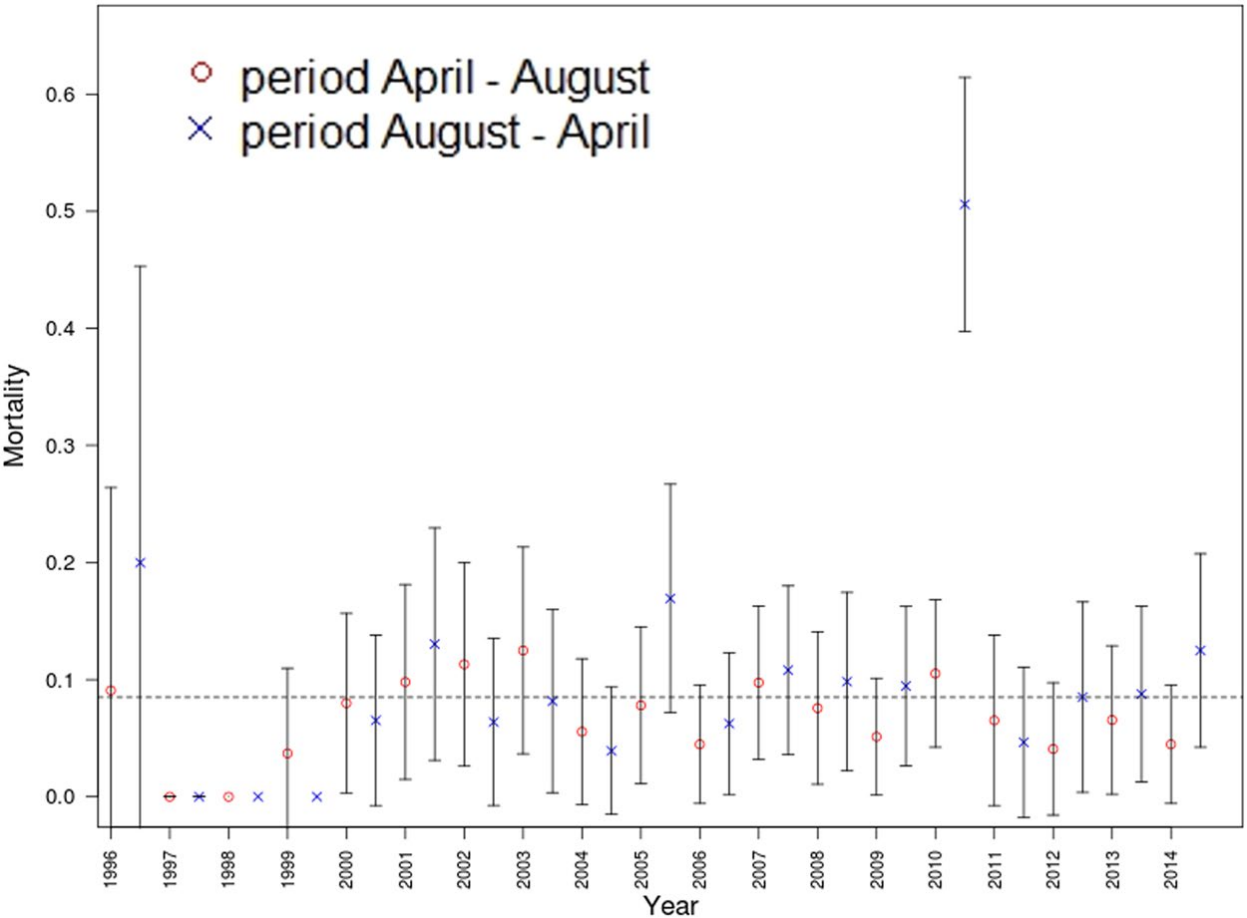
Mortality in ‘summer’ and ‘winter’ periods (±2 s.e.).

Incorporating *year* and *season* (with and without interactions) led to conflicting results. The reasons for these discrepancies became obvious when we looked at Fig. 4, which displays the estimated raw probabilities of death across the 38 analysed seasonal periods. The figure clearly shows that the winter of 2010/11, during which mortality was very high, was responsible for the effects of *year* and *season*. When we statistically controlled for this unusual year by incorporating a factor (*W2010*) representing this particular period, as well as *season*, *year*, and, subsequently, *age* and *size*, again via smooth functions, we no longer observed an effect of season (the results are summarized in Table 2).

**Table 2:** Model comparison for hazard regression for seasonal mortality. *W2010* captures the singular winter period of 2010/11. s*eason* is a two-level factor that distinguishes the summer periods from the non-summer periods. All other variables as in Table 1.

Model	Terms	edf	Deviance	AIC
1	intercept only	1	1245.59	1247.59
2	*W2010*	2	1148.1	1152.1
3	*W2010* + *year*	20	1124.15	1164.15
4	*W2010* + *season*	3	1146.9	1152.9
5	*W2010* + *s*(*age*)	6.02	1140.86	1152.9
6	*W2010* + *s*(*size*)	4.38	1138.03	1146.79
7	*W2010* + *s*(*size*) + *s*(*age*)	8.36	1130.52	1147.24

In the final model, selected for its minimal AIC value, only the impact of the winter season 2010/11 (1 df, *p*-value < 10^−16^) and forearm length (2.383 edf, *p*-value 0.0305) remained as covariates. The age dependence of mortality was again not supported (see also details in Supplementary Material). The impact of the winter period 2010/11 was dramatic: the estimated parameter was =2.44 (s.e. 0.24), which corresponded to a risk of death that is approximately 12 times higher than in the other periods. The model showed that an animal with the median forearm length of 43 mm had a risk of death of 0.486 (s.e. 0.056) in winter 2010/11, but of only 0.076 (s.e. 0.008) in the other periods.

**Figure 5:**
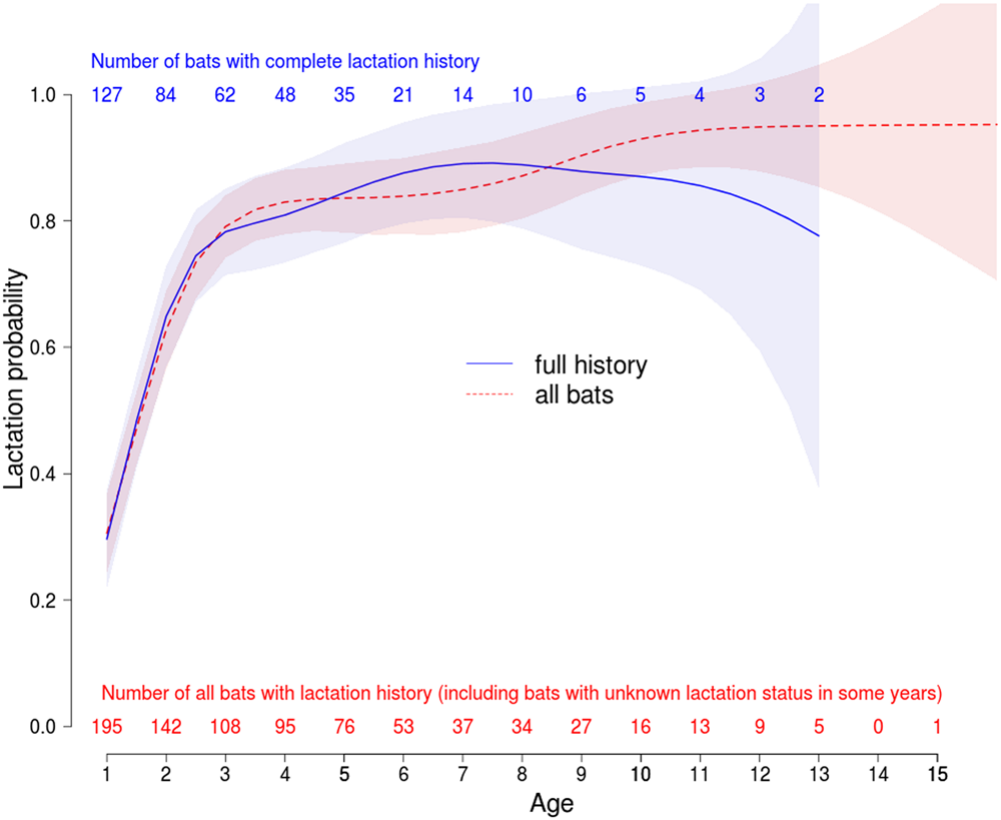
Age-specific fertility as the age-specific probability of lactation during the study period from 1996–2014. The dashed line resembles all bats, including those with gaps in their fertility-history. The number of bats in each age class is shown below the curves in red (n all bats) or above the curve in blue (bats full history). The solid line shows the fit for all bats with a complete fertility-history and the corresponding number of individuals for each age class is given above the solid line.

Age-specific fertility measured as the probability of having lactated at a given age increased steadily until age 3, after which it remained stable at a level of about 0.8 (Fig. 5) regardless of whether we used the dataset with only fully observed individuals, or the full dataset. There was no sign of a decrease in lactation probability with age. Unlike mortality, colony membership had a significant effect on lactation probability. We therefore plotted lactation probability separately for the two colonies with fully observed individuals that could be analysed for this factor. Again, there was no sign of a decrease of lactation probability with age (see supplement).

We calculated generation time T_b_ as 7.33 years for the bats in our study population. Comparing this value to the 15 species of mammals and birds from Jones *et al*. 2008, Bechstein’s bats grouped with much larger animals like the mute swan, red deer and the bighorn sheep in terms of (ln) generation time.

## Discussion

The most important factor that contributed to mortality among Bechstein’s bats in our study was the winter of 2010/11, when death was twelve times more likely than in the other periods. Population breakdowns in the same winter were also reported for Natterer’s bat (*Myotis nattereri*) in the Netherlands, Belgium, and Poland based on the monitoring of hibernacula^34^. It therefore seems likely that severe weather events on a European scale, rather than micro-climatic factors restricted to our study population, were responsible for the excess mortality in 2010. However, we do not know which specific weather conditions led to the devastating effects of this particular winter.

Extreme weather events with excess mortality have been linked to strong selection^35^. A commonly cited example of this association is the change in the beak and the tarsus length of Darwin’s finches during the extreme El Niño in 1977^36^. Despite the high mortality rate in 2010 this did not happen in our study, as shown in Fig. 1. It shows that body sizes of all adult females in our study population remain stable over the entire observational period. In line with this, the mortality-size pattern of 2010 did not differ from that of years with normal mortality levels, such as 2000, which was taken as the reference year (Fig. 3). Large body size was found to be a risk factor for mortality in 2010, and over the whole study period. It should be noted that if we had looked at size-specific mortality in 2010 only, we would have interpreted this extraordinary mortality in 2010 as being a single selective event in which the larger bats suffered higher mortality. Only by looking at the size-mortality relationship over several years were we able to show that larger bats suffered higher mortality in all study years.

Overall, the yearly survival across all age classes in our study population was 83% (se 4%), which increases to 85% (se 2%) after controlling for the winter of 2010. These values are slightly above survival rates reported by previous studies on a number of other temperate bat species with survival rates between 70% and 80%^21, 22, 24, 37^. Within vertebrates, these annual survival values come close to those of large mammals like *Ovis canadensis* ^38^, or non-passeriform birds such as *Larus argentatus* ^39^.

Most importantly, we could not find a significant effect of age on mortality and on lactation probability in our study population. The female adults exhibited no signs of increased mortality with increasing age until they were at least 11 years old and we found no decline in the reproductive rate even in the oldest individuals up to an age of 15 years. *M. bechsteinii* therefore clearly groups with birds that also show constant low mortality over age^2, 14^.

The bats’ mortality was also not affected by season, when the winter of 2010/11 was controlled for. The winter season, as defined in our study, lasted for eight months, including the late summer migration to the swarming site for mating, the hibernation phase, and the spring migration back to the summer habitat. Yet in line with the findings of other bat studies, our results showed that mortality risk did not differ between winter and summer^21, 33^, when controlling for the unusual winter 2010/11. Among birds, by contrast, winter mortality is considerably higher than mortality during the breeding season, regardless what the migratory strategy might be. Residents, partial migrants, and long-distance migrants all have high mortality in the winter season in birds^40^. The same applies to small-sized mammals, but it does not apply to hibernators, which generally have low mortality during hibernation^41^. An exception of this rule is the recent outbreak of the white-nose syndrome in North America killing more than 6 million bats during hibernation within one decade^42^.

Our results confirm the assumption that Bechstein’s bats, as an example for other hibernating Vespertilionid bats with a similar ecology should be placed at the ‘slow’ end of the slow-fast life history continuum, despite their small body size. We have shown that, except during a single catastrophic event, the annual survival rate of the bats was exceptionally high, and that at least throughout the first 11 years of life the bats’ mortality did not depend on age. We conclude that the high adult survival rate among the Bechstein’s bats observed was the main contributor to the growth of their population^16^. Because of their long adult lifespan, the reproductive events of the bats may be dispersed over the entire lifespan. Such a life history strategy is, however, doomed if extreme (e.g., climatic) events or mass killings through pathogenic agents such as the white-nose syndrome, affect the survival rate of the adults. If this survival rate is compromised, more resources must be allocated in current at the expense of future reproduction^43^. If taken to the extreme, this would lead to a semelparous strategy, which is the optimal strategy in extreme environments. Because of their low reproductive output, Bechstein’s bats and most other bat species cannot adopt such a life history strategy and thus are vulnerable to extreme events. Thus, if as predicted, the frequency of extreme events increased due to global climate change^44^ or if novel diseases such as the white-nose syndrome hit populations, this species and other bat species with similar life histories will face a very high extinction risk.

We showed for the first time in long-lived bats that mortality in wild adult females is strongly affected by rare stochastic events, rather than by age, body size, or seasonality. We also found that with respect to age-specific mortality, Bechstein’s bats group with birds rather than with terrestrial mammals, showing no senescence in terms aging and reproduction until high age. Our results have strong implications for the conservation of bats, because they indicate that extreme events may severely disrupt the population dynamics of bats. If those population crashes are related to extreme weather events, an increase in the frequency of such events—as is predicted by climate change models—will represent a severe risk even to currently viable bat populations.

## Methods

### Study site, and data collection

Female Bechstein’s bats from four colonies (BS, GB2, HB, UA)^27, 28^ were monitored in their day roosts (bat boxes) in forests near Würzburg, Germany from 1996 until 2014. All of the adult females (individuals that had survived their first hibernation) were individually marked with small subcutaneously implanted radio-frequency identification (RFID) tags^26^ in all four colonies. In some studies, substantial RFID-tag loss has been recorded^45^, which could lead to an overestimation of mortality if it remains unnoticed. In our study, all individuals were genotyped^28^. Therefore, we could identify individuals that had lost their tags and thus had been marked a second time. This way we could ensure that those bats did not enter our analyses twice. Moreover, RFID-tag loss was below 5%. From mid-April to September/October the presence/absence of individuals was recorded on an almost daily basis using hand-held and automatic RFID-readers attached to the bat boxes occupied by the bats. In addition, in May and in August/September of each year we captured all of the bats present in the four colonies in order to obtain the biometric measurements of each individual and to assess reproductive status. Bats were classified as ‘lactating or post-lactating’, and thus ‘reproduced successfully’ if their teats were extended and bare skin was visible in their vicinity at the end of the lactation season in August^46^. As a measure of body size, we used the animals’ forearm length after their first hibernation, when they were fully grown. Note, that we did not use body mass [g] as an indicator of body size as it turned out to be an unreliable measurement. Body mass strongly fluctuated over the course of a day, as insectivorous bats can consume of up to 30% of their own body weight in a single feeding bout^47^. The bats were marked with an individual RFID tag typically in May following their first hibernation, with the bats that were born the previous year still identifiable at this point by their gray chin spot^31^. Previous population genetic data showed that first-year bats were offspring of the local colonies where they had been found^28, 31^. We therefore calculated recruitment as the number of first-year bats in year x divided by the number of adult females present in year x-1.

Previous research has shown that female Bechstein’s bats are highly philopatric, and thus typically stay in their natal colony throughout their entire life. Very little exchange between colonies has been recorded^28, 31^. The possibility of dispersal could therefore be excluded for the bats that disappeared from our study sites, and a recapture rate of almost 100% per year was achieved (i.e., almost no individuals were missing in a given year and later reappeared). The resulting dataset contains information on 248 adult females of known ages that had survived beyond their first hibernation.

Handling and tagging of the bats were conducted under permits for species protection (55.1-8642.01-2/00) and animal welfare (55.2-2531.01-47/11 and 55.2-2532-2-20) that had been issued by the government of Lower Franconia. All experiments were performed in accordance with relevant guidelines and regulations mentioned above.

### Statistical Analyses

We used discrete-time survival analysis^48^ to study the impact of age, environment and individual size on mortality. Mortality is quantified as the risk of death at age *t* given survival up to *t*, possibly altered by covariates *x* = (*x*_1_, … *x*_*p*_)′. In a first step of analysis age (and calendar time) are considered on an annual basis, starting in the middle of April, when the bats return from their hibernacula^26^. For the (discrete) random variable *T* denoting lifespan (after first hibernation) the hazard is hence defined as,

1 $$h(t|x)=P(T=t| T\ge t,x)$$

where *x* are the covariates that modulate the age-specific risk of death *h*(*t*|*x*). Here, age *t* runs from *t* = 1 to the maximum observed age *L*, which in this dataset was *L* = 15 years. As a specific model for hazard regression we chose a logit link, so that

2 $$logith(t|x)={\alpha }_{t}+{x}^{{\rm{^{\prime} }}}\beta .$$

The parameters *α*_*t*_ describe the age dependence of the hazard (often called the baseline hazard), while the parameters *β* = (*β*_1_, .., *β*_*p*_) capture the influence of the covariates in *x*. As additional covariates, we considered colony; calendar year as a representation of the fluctuation of environmental conditions; and individual size, as measured by an animal’s forearm length. To capture potential nonlinear effects of age *t* and of size parsimoniously, we modeled their impact via smooth functions. All analyses were performed in R version 3.2.3^49^, with the functions gam (R-library mgcv^50^) used to analyse generalized linear and additive models (containing smooth terms^51^), respectively. The models were compared through the analysis of deviance (for nested models) and the Akaike information criterion (AIC^52^).

In a second step, we divided the annual time periods into two ‘seasons’. During the summer period, which ran from 16 April to 15 August, the bats were in the summer habitat where they reproduced. The non-summer period ran from 16 August to 15 April. This period covers the late summer migration from the summer habitat to the swarming sites for mating, the hibernation phase, and the spring migration back to the summer habitat. We chose 16 August as the threshold because this date marks the beginning of the time period when the bats would start to leave the area of the summer roosts to gather at the swarming sites outside the study area^53^. The two seasonal periods differ in length (four vs. eight months), but also cover very different behaviours in the annual cycle that might be associated with rather distinct risks. In this context, our main goal was to investigate whether there were consistent differences in mortality levels between the two periods of the year that are comparable to the seasonal differences in mortality levels in bird populations, among which a peak in mortality during winter has been observed^40^.

Fertility was scored each year a bat was alive as ‘0’ if the bat was caught at the end of the lactation season and has been scored as ‘did not reproduce’, as ‘1’ if it was caught and had been scored as ‘had reproduced’, or as ‘NA’ if the bat was either not caught, or if lactation status was unclear. Due to the relatively high number of observations where reproductive status in a year was unknown (31% of 612 observations) we restricted the dataset to individuals that had a fertility history without ‘NA’s (127 out of 248 individuals, from two of the four colonies). But we will also show the analysis where all observations are included. Because an individual contributed several fertility events, we used a generalized additive mixed model (R-library mgcv^50^) with Bat-ID as random factor.

For comparison with the animal species mentioned in Jones *et al*.^54^ and Gaillard *et al*.^13^, we calculated generation time as the weighted mean age of reproducing females (=T_b_ sensu^55^).

### Data accessibility

Dataset available on request.

## Electronic supplementary material

Supplementary Information
