# Supplementary material for: Rare catastrophic events drive population dynamics in a bat species with negligible senescence
Source: Sci Rep. 2017 Aug 4;7:7370. doi: 10.1038/s41598-017-06392-9 (PMC5544728; doi:10.1038/s41598-017-06392-9)
Supplement: Supplementary file 1 — Supplementary Information [file 41598_2017_6392_MOESM1_ESM.pdf]

Rare catastrophic events drive population dynamics in a bat species with negligible senescence

Toni Fleischer[1,3*], Jutta Gampe[2], Alexander Scheuerlein[3], Gerald Kerth[1]

[1]Applied Zoology and Conservation, Zoological Institute, University of Greifswald, Johann, Sebastian Bach-Strasse 11/12, 17487 Greifswald, Germany

[2]Statistical Demography, Max Planck Institute for Demographic Research, Konrad-Zuse-Str. 1 D-18057 Rostock, Germany

[3]Evolutionary Biodemography, Max Planck Institute for Demographic Research, Konrad-Zuse-Str. 1 D-18057 Rostock, Germany

[*]fleischer@demogr.mpg.de

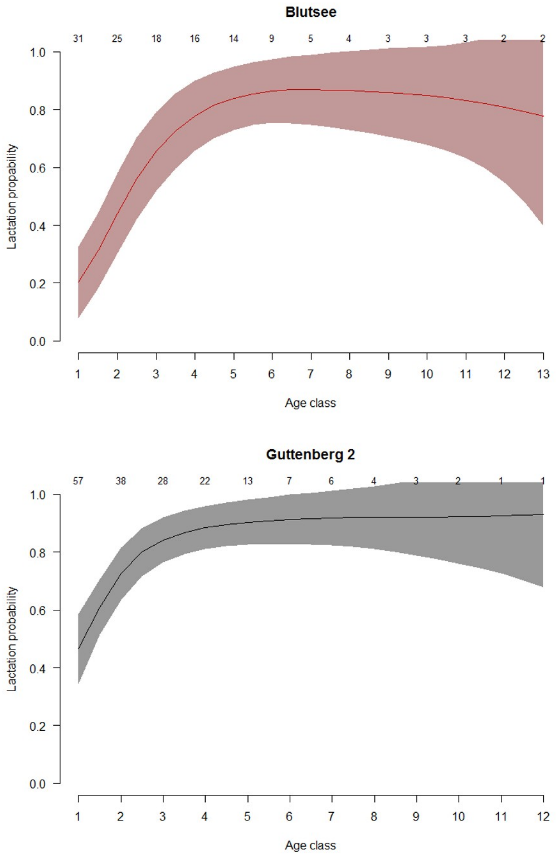

Supplement1: Age dependent fertility for two out of four colonies with full dataset. Like the global trend, the probability to lactate increases within the first three years, where it stays stable until later ages. At the top the number of individuals for each age class are given.
